# Signet ring cell carcinoma and poorly differentiated adenocarcinoma in a gastric hyperplastic polyp with lymphatic invasion: A case report

**DOI:** 10.1016/j.ijscr.2019.10.019

**Published:** 2019-10-17

**Authors:** Kazuya Takahashi, Shin Saito, Yuki Kaneko, Shiro Matsumoto, Hironori Yamaguchi, Joji Kitayama, Yoshinori Hosoya, Hirotoshi Kawata, Alan Kawarai Lefor, Naohiro Sata

**Affiliations:** a Department of Surgery, Jichi Medical University, 3311-1 Yakushiji, Shimotsuke, Tochigi, 329-0498, Japan; b Department of Pathology, Jichi Medical University, Shimotsuke, Japan

**Keywords:** *H. pylori*, *Helicobacter pylori*, Gastric hyperplastic polyp, Diffuse gastric adenocarcinoma, Lymphatic invasion, Helicobacter pylori, Malignant transformation

## Abstract

**Introduction:**

Gastric hyperplastic polyps are common stomach lesion and these polyps are generally benign. However, they can undergo malignant transformation. Most reported cases of malignant transformation of gastric hyperplastic polyps have been to well or moderately differentiated adenocarcinoma, and those transformed into poorly differentiated adenocarcinoma are extremely rare. No case has been reported that has changed to diffuse type adenocarcinoma with lymphatic invasion.

**Presentation of case:**

A 48-year-old woman presented with worsening anemia. A polyp was seen in the gastric cardia seven years prior to presentation. *Helicobacter pylori* infection was also found at that time. She underwent upper gastrointestinal endoscopy and biopsy of the polyp revealed signet ring cell carcinoma. Total gastrectomy was performed due to concern about possible invasion into the submucosal layer and there was no evidence of distant metastases. Histologic examination revealed both poorly differentiated adenocarcinoma and signet ring cell carcinoma surrounded by hyperplastic epithelium at the head of the polyp. Lymphatic invasion was also found, and malignant cells were limited to the mucosa.

**Discussion:**

Gastric hyperplastic polyps are commonly associated with chronic gastritis which is related to *Helicobacter pylori* infections. Gastric hyperplastic polyps are generally benign and rarely undergo malignant transformation to adenocarcinoma with differentiated histology. The gastric hyperplastic polyp in this patient transformed to poorly differentiated adenocarcinoma with lymphatic invasion.

**Conclusion:**

Even small polyps may become poorly differentiated adenocarcinoma with invasion, so close follow-up or endoscopic resection are recommended as well as eradication of *Helico Pylori* infection when appropriate.

## Highlights

• Gastric hyperplastic polyps can turn into adenocarcinoma.
• Very rarely they change into poorly differentiated adenocarcinoma.
• There have been no reports of these polyps transformed into poorly differentiated adenocarcinoma with lymphatic invasion.

## 1 Introduction

Hyperplastic polyps are the most common type of gastric polyps [1] and are usually asymptomatic [2]. Upper gastrointestinal endoscopy is the primary diagnostic modality to identify gastric polyps [3]. It has been reported that gastric hyperplastic polyps are relatively more frequent than fundic gland polyps in regions where *Helicobacter pylori* (*H. pylori*) infection is common [4]. *H. pylori* infections are associated with peptic ulcer disease and neoplasms of the stomach [5]. The inflammation and secreted factors derived from *H. pylori* infections have also been suggested to play a role in the development of gastric hyperplastic polyps [6]. The incidence of *H. Pylori* infection can lead to geographic differences in the prevalence of gastric polyps [7]. Gastric hyperplastic polyps are histologically characterized by dilated, elongated, tortuous foveolar structures lined by hyperplastic gastric mucin-containing epithelium [6]. In general, these polyps are benign [8,9]. However, they can undergo malignant transformation. Previous reports described that the incidence of malignant transformation is 1.5–4.5% [10,11]. Little is known about molecular alternations or pathways associated with malignant transformations of gastric hyperplastic polyps [12]. Most reported cases of malignant transformation of gastric hyperplastic polyps have been to well- or moderately-differentiated adenocarcinoma, and those transformed into poorly differentiated adenocarcinoma are extremely rare [8]. To the best of our knowledge, this is the first report of a patient with both poorly differentiated adenocarcinoma and signet ring cell carcinoma with lymphatic invasion arising in hyperplastic polyp of the stomach. This work is reported in line with the SCARE criteria [13].

## 2 Presentation of case

**Fig. 1:**
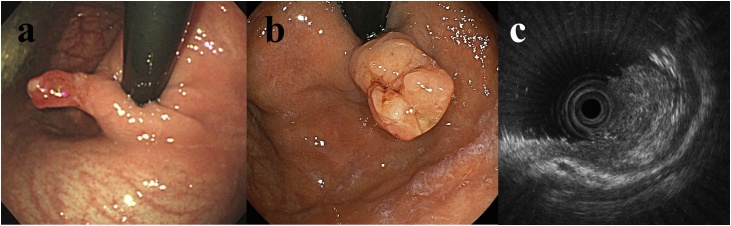
(a) Findings at upper gastrointestinal endoscopy seven years prior to this presentation. There is a 10 mm polyp in the gastric cardia. Biopsy was not performed based on its benign appearance (b) Findings at the latest endoscopy. A polyp with an irregular surface and depressed appearance at the top was found in the same area of the stomach. (c) Endoscopic ultrasound revealed possible invasion into the surface of submucosa.

A 48-year-old woman presented for the workup of anemia. Her medical history included hypertension. Her regular medications were antihypertensives and oral iron preparations. Seven years previously, she underwent upper digestive endoscopy which showed a 10 mm polyp in the gastric cardia (Fig. 1a). Biopsy was not performed at that time because the polyp grossly appeared benign.

She presents now with worsening anemia, documented by a hemoglobin level of 5.6 g/dl. *H. Pylori* antibody was positive, and she underwent upper gastrointestinal endoscopy to evaluate gastrointestinal bleeding as the cause of anemia. A polyp was again seen in the gastric cardia and had increased in size (Fig. 1b). Biopsy of the polyp showed signet ring cell carcinoma. Endoscopic ultrasonography suggested tumor invasion into the surface of submucosa (Fig. 1c). Deeper invasion to the submucosal layer was suggested by the depressed appearance of the polyp (Fig. 1b) and pathological findings of signet ring cell carcinoma. Enhanced computed tomography scan did not show lymphadenopathy or evidence of distant metastases and also showed cholecystolithiasis, adenomyosis of the uterus, and a right ovarian cyst. Adenomyosis of the uterus was considered as the cause of the severe anemia.

**Fig. 2:**
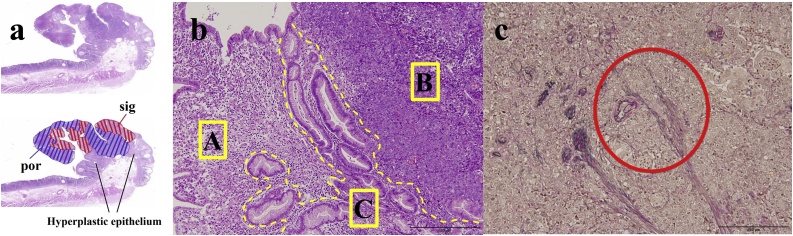
(a) The head of the polyp is replaced by adenocarcinoma. Both signet ring cell adenocarcinoma and poorly differentiated adenocarcinoma are surrounded by hyperplastic epithelium (Loupe image, Hematoxylin and eosin stain). (b) Signet ring cell carcinoma component (A), poorly differentiated adenocarcinoma (B) and hyperplastic epithelium (C) are seen (×100, Hematoxylin and eosin stain). (c) Lymphatic invasion is shown by Elastica van Gieson stain. The red circle shows a vessel filled with tumor cells (×400, Elastica van Gieson).

Total gastrectomy with lymph node dissection, Roux-en-Y anastomosis, cholecystectomy, total hysterectomy, and right adnexa resection were performed. The operation was performed by skilled surgeons. A 11 × 10 × 7 mm 0-I type tumor was found in the cardia of the resected stomach. The specimen revealed both signet ring cell carcinoma and poorly differentiated adenocarcinoma surrounded by hyperplastic epithelium in the head of the polyp (Fig. 2a, b). Although the carcinoma was limited to the mucosal layer, lymphatic invasion was found on the Elastica van Gieson stain (Fig. 2c). She was discharged after an unremarkable postoperative course 14 days after operation.

## 3 Discussion

Gastric hyperplastic polyps occur more frequently in regions with a high prevalence of *H. pylori* infection [14], while the incidence of gastric hyperplastic polyps has decreased in western countries [4]. Gastric hyperplastic polyps almost never occur in normal gastric mucosa and are most commonly associated with chronic gastritis [6]. The presence of *H. pylori* infection is closely related to chronic gastritis which significantly increases the risk of developing peptic ulcer disease, gastric adenocarcinoma and gastric mucosa-associated lymphoid tissue lymphoma [15]. In general, gastric hyperplastic polyps are related to *H. pylori* infections of the stomach [1]. The major risk factors for gastric hyperplastic polyps harboring neoplasms include patient age and polyp size and lobulation [12]. However, chronic *H. pylori* infection plays a central role in the development of gastric cancer as shown by biological and epidemiological studies [16]. Although the relationship between *H. pylori* infection and malignant transformation of gastric hyperplastic polyps is unclear, about 74.7–89.0% of cases of gastric cancer are related to *H. pylori* infections [17].

Gastric cancer can be divided into diffuse or intestinal types based on its histological appearance [18]. Gastric cancer can arise in hyperplastic polyps and a malignant lesion is thought to originate from a hyperplasia-dysplasia-adenocarcinoma sequence [7]. This sequence may contribute to progression of adenocarcinoma associated with intestinal types. Most published cases of gastric cancer originating in hyperplastic polyps have been intestinal-type adenocarcinoma (well or moderately differentiated adenocarcinoma) [8]. The present patient developed a hyperplastic polyp with transformation to both signet-ring cell and poorly differentiated adenocarcinoma (diffuse types). This transformation is extremely rare, and the relationship between dysplasia and diffuse type adenocarcinoma is not clear. To the best of our knowledge, this is the first report of early signet ring cell carcinoma and poorly differentiated adenocarcinoma accompanied by lymphatic invasion arising in a gastric hyperplastic polyp.

Hyperplastic polyps have been reported to regress after eradication of *H. pylori* [19]. Endoscopic resection should be considered for large lesions which may undergo malignant transformation [9,12]. Neoplastic areas of gastric hyperplastic polyps show a loss of p16 and an increased Ki-67 labeling index [12]. These findings may help predict malignant transformation of gastric hyperplastic polyps after biopsy. Resection of gastric hyperplastic polyps larger than 10 mm is recommended [20]. A biopsy of the polyp at the first endoscopy and eradication of *H. Pylori* should have been performed in this patient. The potential for malignant transformation varies among the various types of gastric polyps [3], suggesting that size, *H. pylori* infection status and histologic diagnosis must be considered to guide their management.

## 4 Conclusion

We experienced both early signet ring cell carcinoma and poorly differentiated adenocarcinoma arising in a gastric hyperplastic polyp accompanied by lymphatic invasion. Even small polyps may become poorly differentiated adenocarcinoma with invasion, so close follow-up or endoscopic resection are recommended as well as eradication of *H. Pylori* infection when appropriate.

## Funding

All authors have no funding regarding this paper.

## Ethical approval

The need for ethical approval for this paper was waived by the committee of Jichi Medical University Hospital.

## Consent

Written informed consent was obtained from the patient for publication of this case report and accompanying images.

## Author contribution

All authors in this manuscript contributed to the drafting of this manuscript. AL helped in drafting the manuscript and interpretation of data. SS, KT, SM and YH performed gastrectomy in this patients. HK studied pathological findings. SS and KT obtained the written informed consent from the patient. NS, JK, YK and AL edited the manuscript. All authors have read and approved this manuscript for publication.

## Registration of research studies

The name of registry is research registry, and the unique identifying number (UIN) we obtained is researchregistry5094.

## Guarantor

Dr. Sata, who is the president of Jichi medical university hospital, is the Guarantor.

## Provenance and peer review

Not commissioned, externally peer-reviewed

## Declaration of Competing Interest

All authors declare no conflicts of interests regarding the publication of this paper.
